# Anticipation of Appetitive Operant Action Induces Sustained Dopamine Release in the Nucleus Accumbens

**DOI:** 10.1523/JNEUROSCI.1527-22.2023

**Published:** 2023-05-24

**Authors:** Jessica Goedhoop, Tara Arbab, Ingo Willuhn

**Affiliations:** ^1^Netherlands Institute for Neuroscience, Royal Netherlands Academy of Arts and Sciences, 1105 BA Amsterdam, The Netherlands; ^2^Department of Psychiatry, Amsterdam University Medical Centers, University of Amsterdam, 1105 AZ Amsterdam, The Netherlands

**Keywords:** dopamine, motivated behavior, nucleus accumbens, operant conditioning, pavlovian conditioning, striatum

## Abstract

The mesolimbic dopamine system is implicated in signaling reward-related information as well as in actions that generate rewarding outcomes. These implications are commonly investigated in either pavlovian or operant reinforcement paradigms, where only the latter requires instrumental action. To parse contributions of reward- and action-related information to dopamine signals, we directly compared the two paradigms: male rats underwent either pavlovian or operant conditioning while dopamine release was measured in the nucleus accumbens, a brain region central for processing this information. Task conditions were identical with the exception of the operant-lever response requirement. Rats in both groups released the same quantity of dopamine at the onset of the reward-predictive cue. However, only the operant-conditioning group showed a subsequent, sustained plateau in dopamine concentration throughout the entire 5 s cue presentation (preceding the required action). This dopamine ramp was unaffected by probabilistic reward delivery, occurred exclusively before operant actions, and was not related to task performance or task acquisition as it persisted throughout the 2 week daily behavioral training. Instead, the ramp flexibly increased in duration with longer cue presentation, seemingly modulating the initial cue-onset-triggered dopamine release, that is, the reward prediction error (RPE) signal, as both signal amplitude and sustainment diminished when reward timing was made more predictable. Thus, our findings suggest that RPE and action components of dopamine release can be differentiated temporally into phasic and ramping/sustained signals, respectively, where the latter depends on the former and presumably reflects the anticipation or incentivization of appetitive action, conceptually akin to motivation.

**SIGNIFICANCE STATEMENT** It is unclear whether the components of dopamine signals that are related to reward-associated information and reward-driven approach behavior can be separated. Most studies investigating the dopamine system use either pavlovian or operant conditioning, which both involve the delivery of reward and necessitate appetitive approach behavior. Thus, used exclusively, neither paradigm can disentangle the contributions of these components to dopamine release. However, by combining both paradigms in the same study, we find that anticipation of a reward-driven operant action induces a modulation of reward-prediction-associated dopamine release, producing so-called dopamine ramps. Therefore, our findings provide new insight into dopamine ramps and suggest that dopamine signals integrate reward and appetitive action in a temporally distinguishable, yet dependent, manner.

## Introduction

Striatal dopamine plays a prominent role in motivated behavior and reward learning. More specifically, activity of the mesostriatal dopamine system is associated with movement (Schultz et al., 1983; Jin and Costa, 2010; Howe and Dombeck, 2016; Syed et al., 2016; Da Silva et al., 2018; Lee et al., 2019), motivational processes including the attribution of incentive salience (Berridge, 2007; Flagel et al., 2011; Salamone and Correa, 2012; Saunders et al., 2018), reward value (Hamid et al., 2016), and the so-called temporal difference reward prediction error (RPE), central to reinforcement learning (Sutton and Barto, 1987; Schultz et al., 1997; Bayer and Glimcher, 2005; Hart et al., 2014). A long-standing question is how these aforementioned functions associated with motivation and learning are integrated into the release of dopamine from neuronal terminals in the striatum (Berke, 2018). Relatedly, it is not fully understood whether these functions depend on one another or whether they govern striatal dopamine-release dynamics independently, distinct in *time* (i.e., RPEs are encoded at a different time point than the motivational drive to pursue a reward) or space (i.e., across different regional domains of the striatum).

Regarding temporal separation of dopamine signal functions, theoretical conceptualizations distinguish slow changes (on the order of minutes) affecting tonic or ambient dopamine concentration in contrast to much faster phasic changes (Grace, 1991). Additionally, a number of studies report so-called ramping changes in dopamine release that are of intermediate speed, on the order of seconds (Lerner et al., 2021). Fast dopamine dynamics are proposed to serve a learning function (i.e., encode RPEs and other related value functions), whereas slow dynamics may be important for motivation (Niv, 2007). Consistent with their intermediate timescale, dopamine ramps are hypothesized to be influenced by a number of variables pertinent to either motivation or learning. These variables include reward expectation and proximity, RPE, state value, and uncertainty (Howe et al., 2013; Gershman, 2014; Guru et al., 2020; Kim et al., 2020; Mikhael et al., 2022). Among these, state uncertainty is assumed to be of particular importance as it is impactful and can theoretically explain the effect of the other variables (Starkweather et al., 2017; Kim et al., 2020; Mikhael et al., 2022).

Regarding spatial separation of dopamine-signal functions, differences in dopamine signaling across striatal regions have been reported extensively, demonstrating that representation of these functions varies by region (Lammel et al., 2011; Willuhn et al., 2012, 2014; Klanker et al., 2017; Menegas et al., 2017; de Jong et al., 2019; van Elzelingen et al., 2022a, b). Although the resulting striatal dopamine landscape is not always consistent between studies, consensus is that midbrain dopamine neurons projecting to the nucleus accumbens core, part of the ventromedial striatum (VMS), participate in both motivation and learning (van Elzelingen et al., 2022b). Thus, the VMS is an optimal target to study the integration of dopamine signals encoding motivated actions required to earn rewards as well as the RPEs associated with these rewards. To do so, we measured dopamine release in the VMS of rats undergoing either pavlovian conditioning (PC) or operant conditioning (OC), using this direct comparison to parse the contributions of reward-related information (e.g., reward-predictive cue) and motivated action to a dopamine signal.

Our results show that the onset of a reward-predicting cue induces an initial rise in dopamine release that is indistinguishable between PC and OC. However, we observe marked differences in the sustainment of this dopamine release between PC and OC during the remainder cue presentation, where the only behavioral difference was that PC rats approached the food magazine and OC animals approached the location where the lever extended after cue offset. Together, our findings identify a ramping anticipation component of dopamine release (which is temporally separated from, yet dependent on, the RPE component) and provide new insights on how learning and motivation functions are integrated in VMS dopamine signals.

## Materials and Methods

### Animals

In these experiments, we used exclusively adult male Long–Evans rats (300–400 g); thus, our findings are limited to males. Animals were individually housed and kept on a reversed 12 h day/night cycle (light on from 20:00 to 08:00) with controlled temperature and humidity. A total of 37 rats underwent surgery and were randomly assigned to either PC or OC experimental groups. Because of a nonfunctional or misplaced fast-scan cyclic voltammetry (FSCV) electrode, 12 rats were excluded from this study, and the final group sizes were *n* = 12 for the PC experiment and *n* = 13 for the OC experiment. The rats were food restricted to 85% of their free-feeding body weight, and water was provided *ad libitum*. The rats underwent one training session per day, consistently at the same time. All animal procedures were in accordance with the Dutch and European laws and approved by the Animal Experimentation Committee of the Royal Netherlands Academy of Arts and Sciences.

### Stereotaxic surgery

Rats were anesthetized using isoflurane (1–3%) and placed into the stereotactic frame. Body temperature was maintained using an isothermal pad. The analgesic Metacam (0.2 mg meloxicam/100 g) was delivered using a subcutaneous injection, and the shaved scalp was disinfected using ethanol (70%). An incision of the scalp, which was treated with lidocaine (100 mg/ml), exposed the cranium at the midline. A craniotomy was drilled, and the dura mater was cleared to unilaterally target the nucleus accumbens core of the VMS (1.2 mm AP, 1.5 mm ML, and −7.1 mm DV) with a chronically implanted carbon-fiber electrode (Clark et al., 2010) made in house. An Ag/AgCl reference electrode was positioned in a separate part of the forebrain. The electrodes were secured with cranioplastic cement, which was anchored to the skull by surgical screws. Rats received a subcutaneous injection of 2 ml saline following surgery and were placed in a temperature-controlled cabinet to be monitored for 1 h. Rats were given 1–2 weeks postsurgery to recover before food restriction, behavioral training, and recordings started.

### Magazine and fixed ratio 1 training

All experiments were conducted in modified operant boxes (32 × 30 × 29 cm, Med Associates), equipped with a food magazine (connected to an automated food pellet dispenser) flanked by two retractable levers with cue lights above these levers, a house light, a white-noise generator, and metal grid floors (Med Associates). Each operant box was surveilled by a video camera. The boxes were housed in metal Faraday cages that were insulated with sound-absorbing polyurethane foam. To habituate the rats to these operant boxes before conditioning and to teach the rats that they could obtain food pellet rewards (Dustless Precision Pellets, 45 mg; Bio-Serv), the PC group received two magazine training sessions and the OC group received one magazine training session followed by a fixed ratio 1 (FR1) training session. During all training and recording sessions described here, the house light was illuminated and white-noise was played at an intensity of 65 dB to mask background noises. During magazine training sessions, 45 pellets were delivered on a variable intertrial interval (VITI) of 90 s (range 70–110 s). During the FR1 training session, the active lever (on the left side of the reward magazine) was extended into the operant box at the start of the session, and each lever press resulted in the delivery of one food pellet. In this session, a maximum of 45 food pellets could be earned.

### Pavlovian and operant conditioning

**Figure 1.**
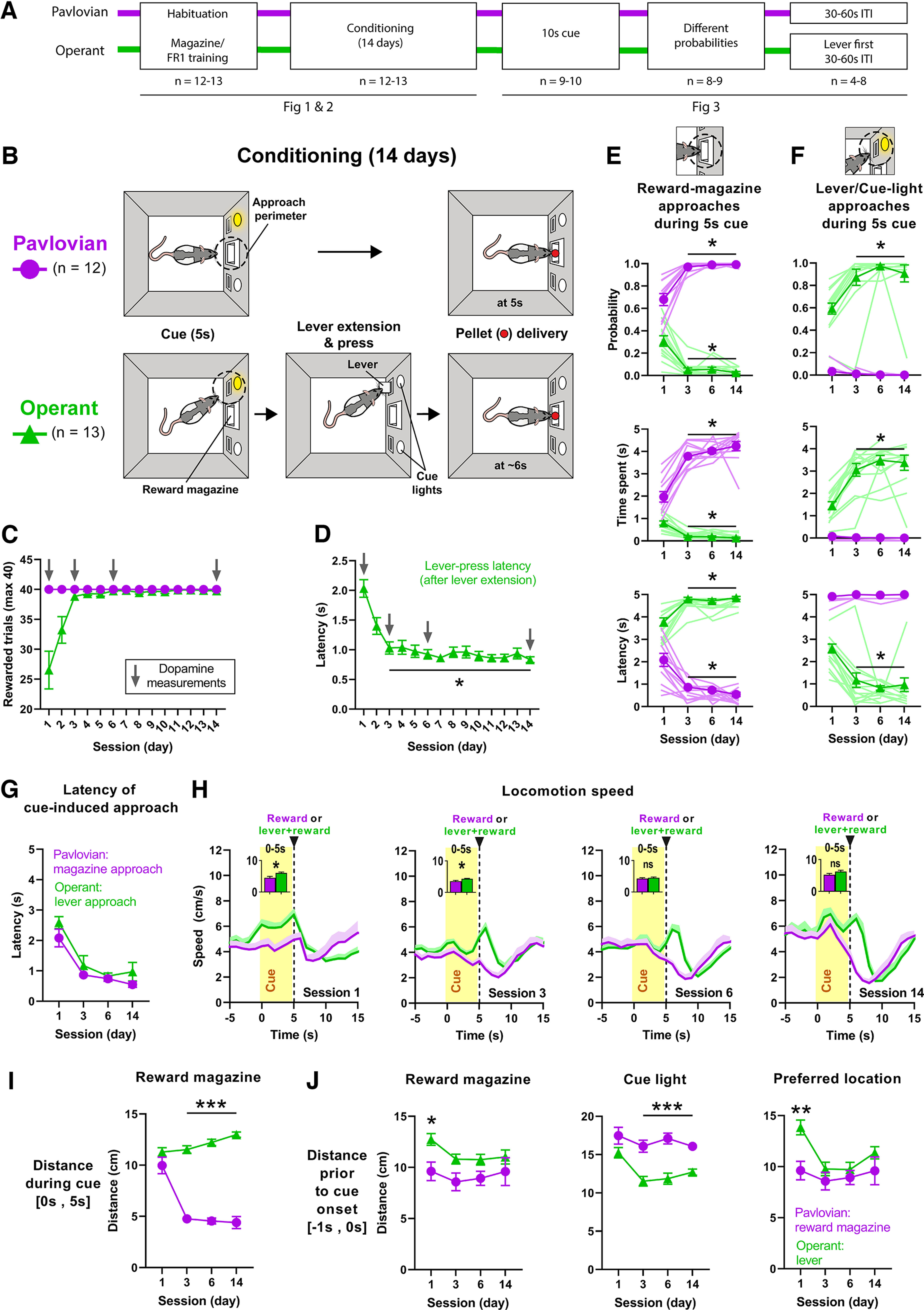
Behavior during pavlovian and operant conditioning. ***A***, Timeline of behavioral training indicating the number of animals per group. ***B***, Schematic of PC and OC tasks. For the PC group (purple circles/traces throughout all figures; *n* = 12), a 5 s cue light exposure was followed by the immediate delivery of a food pellet. For the OC group (green triangles/traces throughout all figures; *n* = 13), a 5 s cue light exposure was followed by the extension of a lever, which needed to be pressed within 5 s for the delivery of a food pellet. ***C***, Average number of rewarded trials (maximum of 40 per session) over the course of conditioning sessions. Arrows mark FSCV-recorded sessions. ***D***, Average latency to lever press after lever extension decreases over the course of conditioning for the OC group. ***E***, Group differences in cue-induced reward-magazine approach are reflected by probability, time spent, and latency during the 5 s cue light. Reward-magazine approach stabilized from session 3 onward. ***F***, Group differences in cue-induced lever/cue light approach behavior are reflected by probability, time spent, and latency during the 5 s cue light exposure (statistics available at https://osf.io/jhz7x/). ***G***, Average latency of the respective cue-induced approach (PC group, toward reward magazine; OC group, toward lever) did not differ between the two groups. ***H***, Average locomotion speed across recording sessions. Insets, Bar graphs depict speed restricted to the 5 s cue-light exposure, which differed significantly between groups during sessions 1 and 3, but not 6 and 14. ***I***, As expected from ***E***, PC rats position themselves closer to the reward magazine during the 5 s cue light presentation (0–5 s). ***J***, Left, However, distance to the magazine just before cue onset (−1–0 s) differs between groups only early in training. Middle, In contrast, OC rats display a shorter distance to the lever/cue light position just before cue onset for most of training. Right, A comparison of the distance to the respective preferred approach location of each group (PC, reward magazine; OC, lever/cue light) just before cue onset reveals a group difference only on day 1. All data are mean + SEM. Single-animal data are represented in lighter-shaded lines in ***E*** and ***F***; *p < 0.05. ns = not significant.

For PC and OC, the rats were placed into the operant boxes and at the start of each conditioning session, the house light was illuminated, the background white noise was turned on, and a VITI of 60 s (range 30–90 s) was initiated. Following the ITI, a cue light was illuminated for a duration of 5 s (Fig. 1*B*). For the PC group, a food pellet was delivered into the reward magazine directly after the cue light turned off, after which the next 60 s VITI started. For the OC group, turning off the cue light was followed by extension of the lever below the cue light into the operant box. The lever was retracted after one lever press (FR1), which immediately resulted in the delivery of one food pellet reward into the food magazine, after which the next 60 s VITI started. If there was no lever press within 5 s after lever extension, the lever was retracted, no reward was delivered, and the next trial was started. The training sessions consisted of 40 trials; after the termination of the session, rats were returned to their home cage.

Rats underwent 22 consecutive daily training sessions during which FSCV recordings took place on days 1, 3, 6, 14, and 22. On day 22 the amount of trials was increased to 80, and the regular trials (described above) were semirandomly intermixed with trials in which an increased reward size was delivered (data not shown) or trials in which the cue light illumination was prolonged to a duration of 10 s. Afterward, a subset of the animals (PC group, *n* = 8; OC group, *n* = 9) was retrained on the regular training schedule for 3 d; on the fourth day, FSCV recordings took place throughout a session consisting of 20 regular trials, followed by 60 trials in which the probability of reward delivery was decreased to *p* = 0.5. A subset of the rats from the OC group (*n* = 8) subsequently received 7 additional days of training with each session consisting of 40 trials in which the contingency was changed. During these sessions the 60 s VITI was followed by immediate extension of the lever, and a lever press (within 5 s of lever extension) resulted in a 5 s cue light illumination, after which the light turned off and a food pellet was delivered. FSCV recordings took place on the seventh day of training on this schedule. During this recording session, a subset of the rats (*n* = 4) received an additional 40 trials in which the probability of reward delivery was decreased to *p* = 0.5. For six PC and seven OC rats, training concluded with 7 d of sessions with a fixed ITI of 30 s, instead of the VITI of 60 s during regular training. These sessions consisted of 40 trials, and FSCV recordings took place on day 7. Figure 1*A* contains a timeline of the behavioral training.

### FSCV measurements and analysis

FSCV was used to detect subsecond changes in extracellular dopamine concentration as described previously (Willuhn et al., 2014). Chronically implanted carbon fiber microelectrodes were connected to a head-mounted voltammetric amplifier, which was interfaced with a PC-driven data acquisition and analysis system (National Instruments) through an electrical commutator (Crist Instrument) mounted above the test chamber. Voltammetric scans were repeated every 100 ms (10 Hz). The electric potential of the carbon fiber electrode was linearly ramped from −0.4 V versus Ag/AgCl to +1.3 V (anodic sweep) and back (cathodic sweep) at 400 V/s (8.5 ms total scan time) during each voltammetric scan, and held at −0.4 V between scans. If present at the surface of the electrode, dopamine is oxidized during the anodic sweep, resulting in the formation of dopamine-o-quinine (peak reaction detected around +0.7 V), which thereafter during the cathodic sweep is reduced back to dopamine (peak reaction detected around −0.3 V). The ensuing flux of electrons is measured as current and is directly proportional to the number of molecules that undergo electrolysis. The background-subtracted, time-resolved current obtained from each scan provides a chemical signature characteristic of the analyte, allowing resolution of dopamine from other substances (Phillips and Wightman, 2003). To isolate dopamine from the voltammetric signal, chemometric analysis with a standard training set was used (Clark et al., 2010). A moving 10-point median filter was used to smooth all data, and baseline subtraction was performed on a trial-by-trial basis before analysis of average concentration. At the start of each FSCV recording session, two unexpected deliveries of a single food pellet (spaced apart by 2 min) confirmed electrode viability to detect dopamine. Animals were excluded from analysis when (1) no dopamine was detected in response to the unexpected pellets before start of the behavioral session, or (2) FSCV recording amplitude background noise was larger than 1 nA in amplitude.

### Behavioral analysis

The delivered rewards and (latency of) lever presses were registered via an automated procedure using Med-PC (Med Associates). DeepLabCut software (Mathis et al., 2018) was used to track the position of the rats in the operant box on video data acquired during FSCV measurements. The tracking data were analyzed using MATLAB (version 2019a, MathWorks) to determine the distance of the rats to the reward magazine and lever (measured from the headcap) and the speed of movement (centimeters/second, measured from the middle of the back). To determine the probability, time spent, and latency of approaching the reward magazine or lever during the cue epoch, approaches were defined by the proximity of the rat to the reward magazine or lever of <5 cm for at least 1 s. The probability of approach in a session was calculated by dividing the number of cue exposures with at least 1 s of approach duration by the total number of cue exposures in a session. Time spent approaching was calculated by averaging the time the rats spent approaching during the cue exposures in a session. The latency of approaching was determined by averaging the latencies of the first approach (of at least 1 s) during the cue exposures in a session. The average locomotion speed was determined during the cue epoch.

### Histologic verification of recording sites

After completion of the experiments, rats were deeply anesthetized using a lethal dose of pentobarbital. Recording sites were marked with an electrolytic lesion before transcardial perfusion with saline, followed by 4% paraformaldehyde (PFA). Subsequently, the brains were removed and postfixated in 4% PFA for 24 h before they were placed in 30% sucrose for cryoprotection. After saturation, the brains were rapidly frozen in an isopentane bath, sliced on a cryostat (50 µm coronal sections, −20°C), and stained with cresyl violet to increase the visibility of the electrode-induced lesions and anatomic structures.

### Statistical analysis

**Figure 2.**
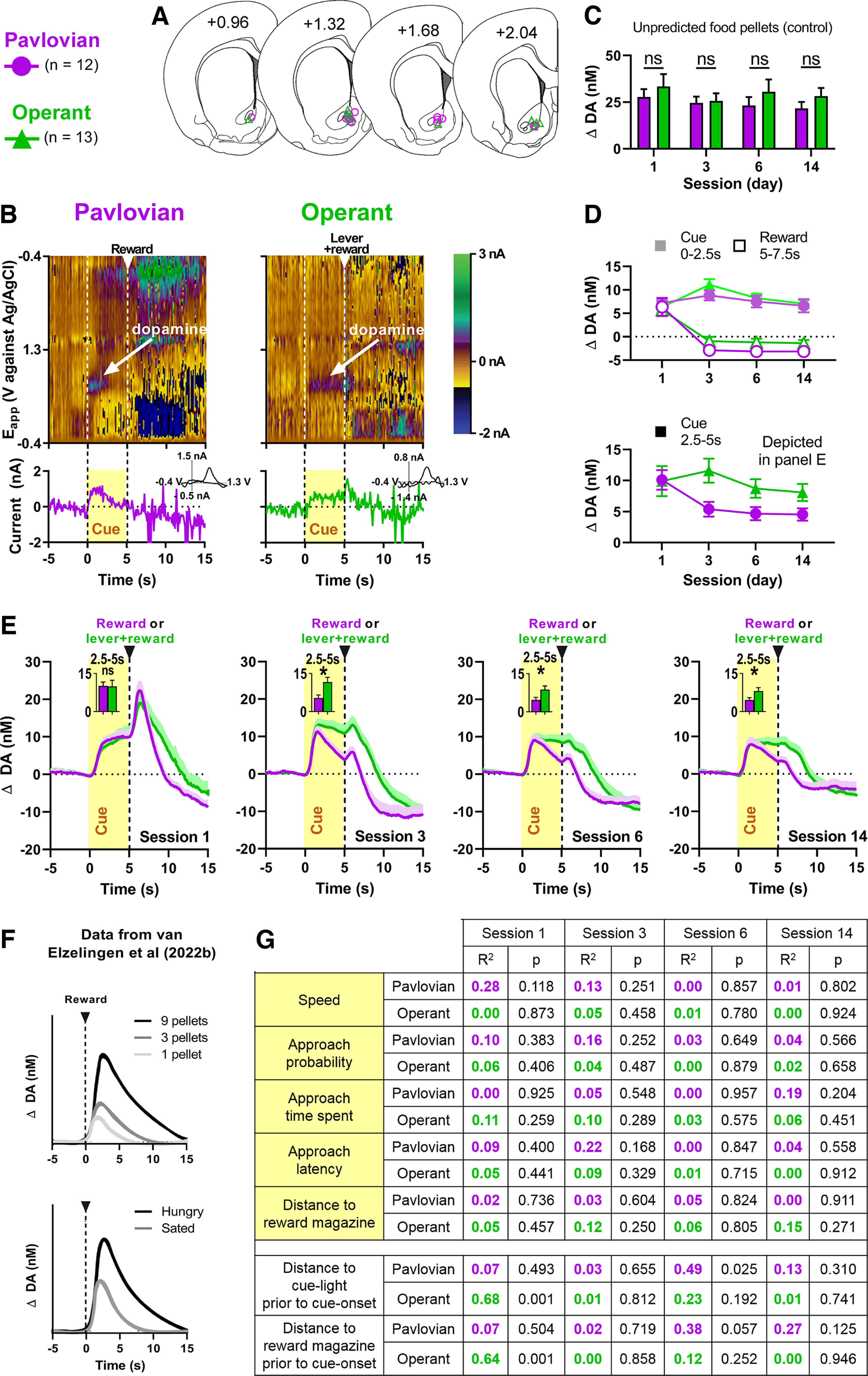
Dopamine release in the VMS during pavlovian and operant conditioning. ***A***, Histologic verification of electrode placement in the VMS. Purple circles represent rats from the PC group (*n* = 12) and green triangles represent rats from the OC group (*n* = 13). ***B***, Example single-trial pseudo-color plots (top) and dopamine traces (bottom) and cyclic voltammograms (insets, bottom) of dopamine recordings in PC (left) and OC (right) groups. Represented are 5 s before cue light exposure, 5 s of cue light exposure, lever press and/or food pellet delivery, and 10 s after the cue light exposure. ***C***, Average peak values of dopamine release in response to unpredicted food pellets. No significant differences were found between PC and OC groups during any of the sessions. ***D***, Top, Average cue-induced (time epoch, 0–2.5 s, baseline set before start cue light exposure) and reward-induced (time epoch, 5–7.5 s, baseline set before food pellet delivery) dopamine release. Bottom, Average cue-induced (time epoch, 2.5–5 s, baseline set before start cue light exposure) dopamine release. ***E***, Average dopamine concentration across seconds (per session). We observed sustained dopamine release during the 5 s cue light in the OC group in all four sessions, in contrast to the PC group, which only showed sustained release during session 1 (time epoch, 2.5–5 s). ***F***, The phasic dopamine peak immediately following a stimulus is known to track changes in reward value and encodes an RPE as dopamine scales with the unexpected delivery of rewards of different value (top, number of food pellets; bottom, hunger state). Modified from van Elzelingen et al. (2022b). ***G***, Top (yellow): There were no correlations between average cue-induced dopamine release (time epoch, 2.5–5 s) and locomotion speed, approach probability, time spent approaching, approach latency, or distance from reward magazine. Bottom (white): There were only sporadic correlations between the distance of the animal to cue or magazine 1 s before cue onset and cue-induced dopamine, and these correlations were not adjusted for multiple comparisons. All data are mean + SEM. Dopamine data are baseline subtracted; *p < 0.05. ns = not significant.

**Figure 3.**
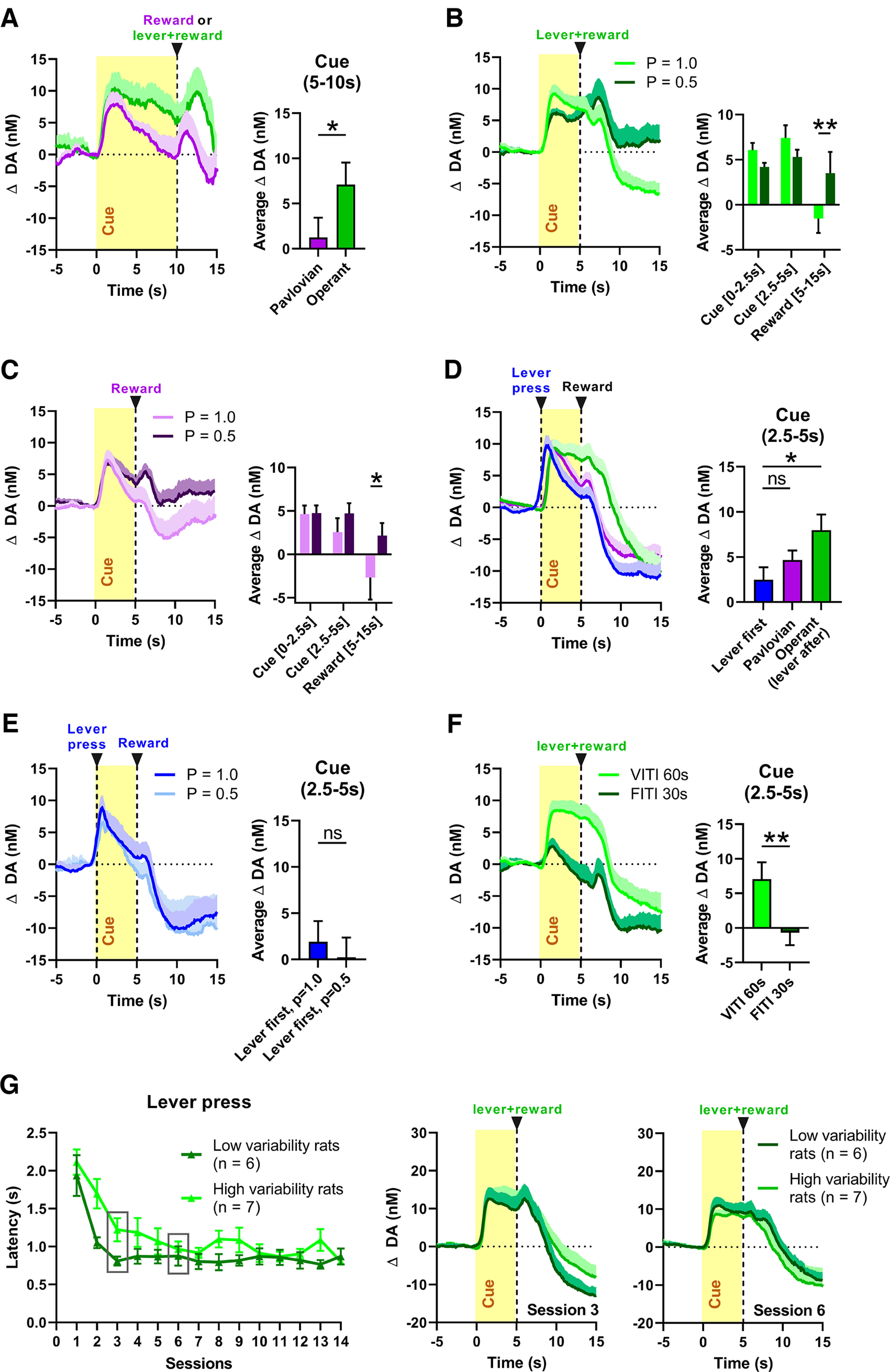
Sustained dopamine release during cue presentation reflects the anticipation of appetitive action. ***A***, Increasing the duration of cue light exposure from 5 s to 10 s prolonged sustained dopamine release (time epoch, 5–10 s) in the OC group (*n* = 10) compared with the PC group (*n* = 9). ***B***, Lowering the probability of food pellet delivery from *p* = 1.0 to *p* = 0.5 did not change sustained cue-induced dopamine release (2.5–5 s) but did increase reward-induced dopamine release (5–15 s) in the OC group (*n* = 9). ***C***, Lowering the probability of food pellet delivery from *p* = 1.0 to *p* = 0.5 did not induce sustained cue-induced dopamine release (2.5–5 s), but did increase reward-induced dopamine release (5–15 s) in the PC group (*n* = 8). ***D***, Requiring a lever press before 5 s of cue light exposure and food pellet delivery (lever-first group, *n* = 8) eliminated sustained dopamine release as cue-induced dopamine release (time epoch, 2.5–5 s) did not differ between the lever-first and PC groups but differed significantly between the lever-first and OC groups. ***E***, Lowering the probability of food pellet delivery from *p* = 1.0 to *p* = 0.5 when a lever press is required before cue and pellet delivery did not affect average cue-induced dopamine release (time epoch, 2.5–5 s; *n* = 4). ***F***, Changing the ITI from a 60 s VITI to an 30 s fixed ITI for the OC group resulted in a significant decrease in average dopamine release (*n* = 7) and no more sustained dopamine release. ***G***, Left, OC rats were divided into two groups, one with low and one with high variance in lever-press latency after cue onset. Dopamine release did not differ between these two groups in session 3 (middle) and session 6 (right), indicating that variable response latency and potentially associated uncertainty were not critical drivers of sustained dopamine. All data are mean + SEM. Dopamine data are baseline subtracted; *p < 0.05, **p < 0.01. ns = not significant.

For all analyses only rewarded trials were included. Behavioral data were analyzed using one- or two-way repeated-measures ANOVAs, unpaired *t* tests, or their nonparametric equivalents when appropriate. *Post hoc* analyses were conducted when necessary and *p* values were adjusted when multiple comparisons were made (except for the regression analyses in Fig. 2*G*). The coefficient of variance was calculated for the latency to lever press using the average latency per session (excluding session 1). Based on this coefficient, a median split divided the OC rats into low-variability and high-variability groups (Fig. 3*G*). Average extra-cellular dopamine concentrations during the last half of the cue epoch (2.5 s in case of a 5 s cue and 5 s in case of a 10 s cue, with baseline set before cue light illumination) and reward epoch (5–7.5 or 5–15 s after cue onset, with baseline set before reward delivery) were compared between groups using unpaired *t* tests or one-way ANOVAs or their nonparametric equivalents when appropriate. For FSCV recordings in which the probability of reward delivery decreased within the session (*p* = 1.0 to *p* = 0.5), we used the average of the last 20 *p* = 0.5 trials of the session in which a reward was delivered (excluding prior trials in which the animals were learning the new contingency). We compared average dopamine during the first half of the cue epoch (0–2.5 s), second half of the cue epoch (2.5–5 s), and the reward epoch (5–15 s) using a two-way repeated measures ANOVA. In case of other within-subject comparisons, a paired *t* test or its nonparametric equivalent was used. Regression analyses were performed to test for correlations between the average locomotion speed, approach probability, time spent approaching, approach latency, average distance from the reward magazine during the cue epoch as well as distance to cue light 1 s before cue onset, distance to magazine 1 s before cue onset, and average dopamine concentrations during the cue epoch.

### Data availability

The data that support the findings of this study are available at https://osf.io/jhz7x/. The code used for this study is available from the corresponding author on request.

## Results

### Behavior during pavlovian and operant conditioning

Rats were trained on a VITI of 60 s using one of two experimental paradigms, either PC, in which a cue signals reward delivery, or OC, in which the same cue signals that a (required) lever press will produce reward delivery (Fig. 1*A*,*B*). PC rats (*n* = 12) received the maximum number of obtainable rewards (40) immediately from session 1 onward, whereas OC rats (*n* = 13) on average earned the maximum number of rewards from approximately session 3 onward (Fig. 1*C*). This learning curve of the OC group was also reflected in the decreasing latency to lever press after lever extension [main effect, χ^2^(14) = 5268, *p* < 0.0001], with sessions 3 through 14 having a significantly lower latency to lever press compared with session 1 (1 vs 3, *p* = 0.0475; 1 vs 4, *p* = 0.0134; 1 vs 5, *p* = 0.0081; 1 vs 6, *p* = 0.0016; 1 vs 7, *p* < 0.0001; 1 vs 8, *p* = 0.0013; 1 vs 9, *p* = 0.0068; 1 vs 10, *p* = 0.0002; 1 vs 11, *p* = 0.0002; 1 vs 12, *p* = 0.0002; 1 vs 13, *p* = 0.0017; 1 vs 14, *p* < 0.0001; Fig. 1*D*). We restricted the following behavioral analysis to those sessions (1, 3, 6, 14) in which dopamine measurements were taken. The two groups differed in their appetitive approach behavior during the 5 s cue light exposure. Over the course of conditioning, the PC group rapidly developed cue-induced approach behavior toward the reward magazine, illustrated by a high probability (main effect of session, *F*_(1.273,28.01)_ = 0.4624, *p* = 0.5481; main effect of group, *F*_(1,23)_ = 1025, *p* < 0.0001; session × group interaction, *F*_(3,66)_ = 68.90, *p* < 0.0001), much time spent (main effect of session, *F*_(2.021,44.45)_ = 19.19, *p* < 0.0001; main effect of group, *F*_(1,23)_ = 742.6, *p* < 0.0001; session × group interaction, *F*_(3,66)_ = 67.94, *p* < 0.0001) and low latency (main effect of session, *F*_(1.599,35.17)_ = 1.249, *p* < 0.0001; main effect of group, *F*_(1,23)_ = 699.0, *p* < 0.0001; session × group interaction, *F*_(3,66)_ = 48.55, *p* < 0.0001) to approach this section of the operant box, whereas the OC group did not (Fig. 1*E*). *Post hoc* analysis revealed that sessions 3, 6, and 14 differed significantly from session 1 in reward-magazine approach probability (PC, 1 vs 3, *p* = 0.0007; 1 vs 6, *p* = 0.0009; 1 vs 14, 0.0012; OC, 1 vs 3, 0.0002; 1 vs 6, *p* = 0.0001; 1 vs 14, 0.0004), time spent (PC, 1 vs 3, *p* < 0.0001; 1 vs 6, *p* < 0.0001; 1 vs 14, *p* = 0.0001; OC, 1 vs 3, *p* < 0.0001; 1 vs 6, *p* < 0.0001; 1 vs 14, 0.0002), and latency (PC, 1 vs 3, *p* = 0.0017; 1 vs 6, *p* = 0.0017; 1 vs 14, *p* = 0.0039; OC, 1 vs 3, *p* = 0.0001; 1 vs 6, *p* < 0.0001; 1 vs 14, *p* = 0.0004); sessions 3, 6, and 14 did not, however, differ significantly from each other, indicating that from session 3 onward, conditioned approach behavior was stable, and no additional learning occurred after this time point. In contrast to the PC group, the OC group rapidly developed cue-induced approach behavior toward the lever below the cue light, which they were trained to press after the cue light turned off to obtain a food pellet. This, too, was illustrated by a high probability (main effect of session, *F*_(1.468,31.81)_ = 14.23, *p* = 0.0002; main effect of group, *F*_(1,23)_ = 303.1, *p* < 0.0001; session × group interaction, *F*_(3,65)_ = 20.50, *p* < 0.0001), much time spent (main effect of session, *F*_(2.183,47.30)_ = 26.93, *p* < 0.0001; main effect of group, *F*_(1,23)_ = 174.7, *p* < 0.0001; session × group interaction, *F*_(3,65)_ = 30.88, *p* < 0.0001), and a low latency (main effect of session, *F*_(1.899,41.15)_ = 16.10, *p* < 0.0001; main effect of group, *F*_(1,23)_ = 345.8, *p* < 0.0001; session × group interaction, *F*_(3,65)_ = 19.33, *p* < 0.0001) to approach the lever during the 5 s cue exposure (Fig. 1*F*). Again, *post hoc* analysis revealed that sessions 3, 6, and 14 differed significantly from session 1 in lever approach probability (1 vs 3, *p* < 0.0001; 1 vs 6, *p* < 0.0001; 1 vs 14, *p* = 0.0003), time spent (1 vs 3, *p* < 0.0001; 1 vs 6, *p* < 0.0001; 1 vs 14, *p* = 0.0002), and latency (1 vs 3, *p* = 0.0003; 1 vs 6, *p* < 0.0001; 1 vs 14, *p* = 0.0004); sessions 3, 6, and 14 did not differ significantly from each other, indicating that in the OC group as well, from session 3 onward, conditioned approach behavior was stable, and no additional learning occurred after this time point. The number of PC group approaches to the lever or cue light did not differ significantly between sessions. Thus, although the two groups approached different areas of the operant box, both groups learned this appetitive approach behavior at the same rate (i.e., by day 3); this is also demonstrated by the latencies of their respective cue-induced approaches, which did not differ between the two groups (main effect of group, *F*_(1,23)_ = 2.140, *p* = 0.1571; Fig. 1*G*). Conceptually, this latency might also reflect approach vigor, which implies that vigor also did not differ between the two groups. To further investigate this last point, we compared the locomotion speed of the groups during the 5 s cue light exposure (Fig. 1*H*). Locomotion speed between PC and OC groups differed significantly in sessions 1 (*U* = 20, *p* = 0.0041) and 3 (*U* = 35, *p* = 0.0188), but not sessions 6 (*U* = 61, *p* = 0.3760) and 14 (*U* = 35, *p* = 0.1072). Together, we conclude that approach vigor overall (measured as approach latency and speed) did not differ between groups.

The groups did not differ in their distance to the reward magazine during the 5 s cue exposure in session 1; however, there was a significant difference in subsequent sessions (Fig. 1*I*) when the PC group had learned to approach the magazine and the OC group had learned to approach the lever instead (main effect of group, *F*_(1,23)_ = 215.5, *p* < 0.0001; main effect of session, *F*_(2.025,44.55)_ = 15.87, *p* < 0.0001; session × group interaction, *F*_(3,66)_ = 32.69, *p* < 0.0001; *post hoc* analysis, session 1, *p* = 0.5320; session 3, *p* < 0.0001; session 6, *p* < 0.0001; session 14, *p* < 0.0001). Because dopamine release can be affected by the distance of the individual to reward location (Howe et al., 2013), we investigated distance to reward magazine during the last second before cue onset (Fig. 1*J*, left). Only the precue distance on day 1 differed significantly between PC and OC rats (main effect of group, *F*_(1,23)_ = 7.294, *p* = 0.0128; main effect of session, *F*_(2.128,46.81)_ = 1.894, *p* = 0.1595; session × group interaction, *F*_(3,66)_ = 0.8846, *p* = 0.4537; *post hoc* analysis, session 1, *p* = 0.0391; session 3, *p* = 0.1405; session 6, *p* = 0.1746; session 14, *p* = 0.8335). We also analyzed the distance to the cue light during the last second before cue onset (Fig. 1*J*, middle) and found significant differences on days 3, 6, and 14 (main effect of group, *F*_(1,23)_ = 32.93, *p* < 0.0001 group; main effect of session, *F*_(2.707,50.53)_ = 7.543, *p* = 0.0004; session × group interaction, *F*_(3,56)_ = 2.433, *p* = 0.0744; *post hoc* analysis, session 1, *p* = 0.3595; session 3, *p* = 0.0007; session 6, *p* = 0.0003; session 14, *p* = 0.0004). However, unlike OC rats, PC rats were not attracted to the cue. Therefore, we also compared the distance to the respective preferred approach location of each group (PC, reward magazine; OC, lever/cue light) during the second before cue onset. Only the precue distance on day 1 is significantly different between PC and OC rats and not during the other days (main effect of group, *F*_(1,23)_ = 6.430, *p* = 0.0185; main effect of session, *F*_(2.414,53.91)_ = 5.247, *p* = 0.0054; session × group interaction, *F*_(3,67)_ = 2.687, *p* = 0.0534; *post hoc* analysis, session 1, *p* = 0.0065; session 3, *p* = 0.7360; session 6, *p* = 0.8882; session 14, *p* = 0.6852; Fig. 1*J*, right). Thus, once animals had acquired their respective approach behavior, they spent most of the time during cue exposure near the approached object and moved away from the food magazine after reward consumption.

### Dopamine release in the VMS during pavlovian and operant conditioning

Extracellular dopamine fluctuations were measured during PC and OC sessions using FSCV, with chronic electrodes targeting the VMS (Fig. 2*A*). Both groups released dopamine in response to cue presentation as shown in the representative color plots in Figure 2*B*. In addition, both groups stably released dopamine in response to the delivery of unpredicted food pellets, given as a control for electrode viability before the start of each session; there was no difference between groups (main effect of time, *F*_(2.618,56.73)_ = 0.8803, *p* = 0.4448; main effect of group, *F*_(1,23)_ = 1.983, *p* = 0.1724; Fig. 2*C*). Average dopamine release during the 5 s cue exposure (time epoch, 0–2.5 and 2.5–5 s, with baseline set before cue light illumination) and reward delivery (time epoch, 5–7.5 s, with baseline set before pellet delivery) remained relatively stable over time (Fig. 2*D*), where dopamine release did not differ between groups at initial cue onset (0–2.5 s) or after reward delivery but did differ during cue exposure (2.5–5 s; see below). More specifically, although dopamine induced by cue exposure (2.5–5 s) did not differ between groups in session 1 (*t*_(21)_ = 0.0612, *p* = 0.9518), it differed significantly in subsequent sessions (3, *t*_(23)_ = 2.686, *p* = 0.0132; 6, *t*_(23)_ = 2.188, *p* = 0.0391; 14, *t*_(20)_ = 2.147, *p* = 0.0443; Fig. 2*D*, bottom, *E*). The dynamics are characterized, respectively, by an initial rise (which is known to track changes in reward value and encodes an RPE; Fig. 2*F*) followed by an immediate drop toward baseline dopamine concentration for the PC group, whereas cue-induced dopamine in the OC group was sustained throughout the 5 s cue exposure (which the PC group showed only in session 1). Reward-induced changes in dopamine because of reward delivery did not differ between groups throughout sessions (1, *U* = 47, *p* = 0.2839; 3, *U* = 59, *p* = 0.3203; 6, *U* = 51, *p* = 0.1519; 14, *U* = 43, *p* = 0.2829; Fig. 2*D*, top). In addition, in neither of the groups was cue-induced dopamine release (time epoch, 2.5–5 s) correlated to cue-induced locomotion speed, approach probability, time spent approaching, approach latency, nor distance to the food magazine in any of the conditioning sessions (Fig. 2*G*). Furthermore, cue-induced dopamine was only rarely and infrequently correlated to the distance of the animals to cue or magazine 1 s before cue onset (Fig. 2*G*; not adjusted for multiple comparisons), in contrast to the consistent dopamine differences across sessions.

To better understand the different dopamine dynamics of the groups during the 5 s cue exposure, several additional training and recording sessions were performed in which we varied task-relevant parameters. For the first experiment, we set out to further investigate how the anticipation to perform an action (the lever press) affects dopamine. We prolonged the duration of anticipation to lever press by increasing the cue light duration to 10 s, which resulted in a significant difference in average dopamine release between groups (time epoch, 5–10 s, with baseline set before cue illumination; *t*_(17)_ = 1.759, *p* = 0.0483), with the OC group (*n* = 10) showing sustained dopamine release for the entire cue period in contrast to the PC group (*n* = 9, Fig. 3*A*).

Further, we hypothesized that OC-group rats may experience greater uncertainty about receiving the food pellet as reward delivery was contingent on lever pressing (despite fulfilling this lever-press requirement correctly in almost all trials from session 3 onward) and that this uncertainty might induce more sustained dopamine release during the 5 s cue exposure compared with the PC group (which had no operant requirement). This idea is supported by the fact that in the PC group cue-induced dopamine release was similarly sustained in session 1, in which the rats were still learning the contingency between cue and reward and, thus, experienced greater uncertainty about receiving reward. We increased uncertainty by decreasing the probability of food pellet delivery from *p* = 1.0 to *p* = 0.5 and compared dopamine release during different epochs, the first half of the cue (0–2.5 s), second half of the cue (2.5–5 s), and reward delivery (5–15 s). In the OC group (Fig. 3*B*), we found no significant main effect of probability (*F*_(1,8)_ = 0.2962, *p* = 0.6011); however, there was a main effect of epoch (*F*_(2,16)_ = 7.495, *p* = 0.0050) and an interaction effect (*F*_(2,16)_ = 12.66, *p* = 0.0005). In the PC group (Fig. 3*C*), we found main effects of probability (*F*_(1.000,7.000)_ = 6.725, *p* = 0.0358) and epoch (*F*_(1.200,8.400)_ = 8.815, *p* = 0.0144), as well as an interaction effect (*F*_(1.455,10.19)_ = 8.002, *p* = 0.0118). *Post hoc* analyses revealed no significant difference in dopamine during the cue epochs in both PC and OC groups (first cue half, PC, *p* = 0.998; OC, *p* = 0.3168; second cue half, PC, *p* = 0.2388; OC, *p* = 0.2239). In contrast, increased uncertainty augmented dopamine release after reward delivery, indicating that rats were learning the new reward probability (PC, *p* = 0.0452; OC, *p* = 0.0013).

In another experiment, we eliminated the element of anticipation to lever press from the 5 s cue light period. We switched the order of appearance of lever and cue light in the OC group so that the 60 s VITI was followed by immediate extension of the lever into the operant box, and a lever press (within 5 s of lever extension) resulted in a 5 s cue light illumination, after which the light turned off and a reward was delivered. As a consequence, rats (lever-first group, *n* = 8) did not show sustained dopamine release during the cue light, and their dopamine dynamics resembled that of the PC group (Fig. 3*D*); the latter observation is supported by the fact that mean dopamine concentration during cue exposure did not differ significantly between lever-first and PC groups conditioned for a similar number of days (main effect, *F*_(2,30)_ = 3.310, *p* = 0.0502; *post hoc* testing, *p* = 0.5101), yet differed significantly from rats that underwent regular OC (lever press required after the cue light turned off; *p* = 0.0338). In a subset of these lever-first rats, we decreased the probability of food pellet delivery from *p* = 1.0 to *p* = 0.5 (*n* = 4), which again did not significantly alter cue-induced dopamine release (*t*_(3)_ = 0.8821, *p* = 0.2213; Fig. 3*E*).

All previously described experiments made use of a VITI of 60 s (range, 30–90 s), thus, rats were not able to predict when in the session the next trial would start (i.e., when the cue light is turned on). In a final experiment, we tested whether such session uncertainty was a prerequisite for the observed effects by using a highly predictable, fixed ITI of 30 s, which eliminated almost all cue-induced dopamine (Fig. 3*F*). No more sustained dopamine release was observed in the OC group, and the average dopamine concentration was significantly lower compared with the 60 s VITI conditions (*n* = 7, *t*_(6)_ = 3.418, *p* = 0.0071).

To explore a potential influence of reward-delivery timing and the associated uncertainty, we calculated the coefficient of variance for the latency to lever press across training sessions, followed by a median split resulting in a low-variability group and a high-variability one. Figure 3*G* depicts the lever-press latencies of these two groups across the 14 d of training (left) and their respective dopamine signals for training days 3 and 6 (right). Day 3 was chosen because group latencies were markedly different, whereas they overlapped on day 6. Our results demonstrate that low- and high-variability groups did not exhibit differences in dopamine release, indicating that specific reward timing cannot account for the occurrence of sustained dopamine release in OC rats.

## Discussion

To investigate how the mesolimbic dopamine system integrates functions related to reward and actions associated with the pursuit of reward, we measured VMS dopamine release in rats undergoing either PC or OC, in which a visual cue signaled either the upcoming delivery of a food pellet or the opportunity to execute an action to obtain this reward, respectively. Initial dopamine-release amplitude to the cue was similar between groups, but in OC we observed a sustained elevation of dopamine concentration subsequently (throughout cue presentation and before lever press) compared with PC. This dopamine sustainment, akin to what has been referred to as a dopamine ramp (albeit not ramping up), was observed reliably and consistently throughout systematic manipulation of experimental parameters and behavioral training, and, thus, we interpret it as associated with the anticipation or preparation to execute the (learned) operant action.

Our parallel PC/OC study design used two paradigms that are nearly identical and differ only by a brief instrumental action in OC (operant-lever response requirement), which takes place in close physical proximity to the food magazine (∼3 cm away) following the offset of the 5 s cue presentation. The onset of the cue induces immediate appetitive approach behavior in both groups of rats, distinguishable only by its target location (OC rats approached the lever site and PC rats the food magazine). During the 5 s cue epoch both groups remain near their respective target location, whereas only after cue offset behavior differed momentarily as PC rats consumed the food immediately, and OC rats performed a brief, single lever press just before food consumption. Both groups acquired the approach behavior with a similar time course, where behavior was already stable after 2–3 of the 14 training sessions. Thus, because overall behavior during cue presentation did not differ between groups, we can exclude a number of explanations for the observed differences in sustained dopamine release. Such differences cannot be attributed to learning as group behavior did not differ in learning rate and dopamine signaling also developed with a similar time course in both groups (two to three sessions). Furthermore, postlearning behavior (extended behavioral training, 14 sessions) during cue presentation was similar in both groups, indicating that approach behavior, task performance, distance to cue or reward magazine, and general speed of movement (Figs. 1*E–H*, 2*G*; including response vigor) were not sources for differential dopamine signaling. Although we found group differences in the position of the animals relative to reward magazine and cue location, these did not correlate with dopamine release and were not consistent with group differences in dopamine release. Location discrepancies before cue onset are especially unlikely to contribute to PC/OC differences in dopamine as initial dopamine-peak size after cue onset never differed between groups. Furthermore, because PC animals did not approach the cue at all but instead approached the reward magazine exclusively, it is also unlikely that sustained OC dopamine was associated with a sign-tracking phenomenon. Finally, differential dopamine dynamics cannot be explained by varying electrode sensitivity, which was stable across training and groups. Together, because of the aforementioned similarity of OC and PC behavior (before lever press), our findings suggest that sustained OC dopamine is related to behavior that has not yet occurred—the upcoming lever press after cue offset.

To further interrogate the nature of sustained dopamine release, we systematically varied behavioral paradigm parameters. First, we extended the duration of the predictive cue from 5 to 10 s, which extended the sustained release until lever press and, thus, demonstrated that this dopamine ramp is flexible in duration and is associated with a state that directly precedes action initiation. Such a state may for example be linked to the readiness to perform an action and may bridge the time between action anticipation and its execution. Next, as previous work demonstrated that the dopamine system is sensitive to reward uncertainty (Fiorillo et al., 2003; Kobayashi and Schultz, 2008; de Lafuente and Romo, 2011; Lak et al., 2017; Starkweather et al., 2017), we rendered food delivery probabilistic (only 50% of trials rewarded vs 100% in previous experiments). The subsequent increase in reward-induced dopamine indicated that rats perceived this change in probability. However, in neither PC or OC did such uncertainty lead to a significant change in sustained dopamine. Furthermore, dopamine release between OC rats with low and high variability in lever-press latency did not differ, indicating that uncertainty (that may be associated with such variable latency) is not a critical driver of sustained dopamine. In contrast, the dopamine ramp disappeared after we moved the lever-press requirement forward in time, from cue offset to cue onset; thus, sustained dopamine is only observed with an operant requirement that follows a period of action anticipation. In this lever-first situation, increasing uncertainty by rewarding only 50% of trials did not reinstate sustained dopamine, further underlining the insensitivity of this ramp phenomenon to uncertainty. Thus, dopamine ramps were not a simple product of reward prediction by the unexpected cue onset (variable ITIs prevent the animals from predicting time of cue onset) and operant requirement but instead a product of the specific sequence of the two, that is, reward-predicting cue followed by lever press. Moreover, this dopamine ramp instantiates a modulation or extension of the initial phasic component of dopamine release (consistent with an RPE signal; Fig. 2*F*; Hart et al., 2014; van Elzelingen et al., 2022b) as both amplitude and sustainment of dopamine ramps diminish drastically when the timing of reward delivery is (more) temporally predictable (fixed ITI). Interestingly, the initial dopamine peak had the same amplitude in both groups, indicating no value discounting (e.g., because of the required effort), and indicating that the ramp did not consist of a redistribution of RPE-related dopamine signaling. Therefore, together, we speculate that the two core elements necessary for dopamine ramps constitute a positive RPE followed by an operant-action anticipation necessary to earn this reward, neither of which was sufficient to produce a dopamine ramp on its own. Uncertainty seems not to affect this ramp; however, we cannot completely rule that out.

Ruling out movement as a direct source for changes in dopamine signaling seems, at first glance, inconsistent with several previous studies. However, most studies that tie the dopamine system to initiation of specific movement, vigor, or velocity were executed on the single-cell level (Jin and Costa, 2010; Puryear et al., 2010; Wang and Tsien, 2011; Barter et al., 2015; Dodson et al., 2016; Howe and Dombeck, 2016; Coddington and Dudman, 2018; Da Silva et al., 2018; Engelhard et al., 2019; Hughes et al., 2020), whereas our study evaluates bulk signal from dopamine neuron terminals, reflecting dopamine released from many neurons, which likely dilutes movement-specific activity of individual neurons. Indeed, bulk signal studies report more general associations with movement (Flagel et al., 2011; Syed et al., 2016; Lee et al., 2019). Notably, the dopamine system is set up anatomically to broadcast its signals to striatal targets via bulk or population signals as dopamine released from a large number of extrasynaptic terminals is pooled, resulting in a diffusion-based signal that is perpetuated by volume transmission (Rice and Cragg, 2008). Furthermore, terminal release can be modified independently of cell-body activity, which may contribute to a discrepancy in findings between sampling dopamine cell bodies and terminals (Threlfell et al., 2012). Relatedly, our results indicate a potential fusion or dependency of RPE and action-anticipation signals, consistent with a report that suggests a dependence of dopaminergic reward processing on movement related to reward pursuit (Syed et al., 2016). But in our case this dependence is inverted; that is, the action-anticipation signal is dependent on the putative RPE signal. Together, our results therefore suggest that unlike single dopamine neuron activity in the midbrain, bulk terminal dopamine release does not encode movement. However, our findings nonetheless suggest a link between dopamine RPE and movement, albeit with a not yet executed, anticipated movement.

So-called dopamine ramps are reported to occur over a timescale of seconds in both dopaminergic cell bodies in the midbrain as well as in their axon terminals in the ventral striatum, often when dopamine neuron activity (including dopamine release) was sensed as bulk activity (Howe et al., 2013; Collins et al., 2016; Hamid et al., 2016; Guru et al., 2020; Kim et al., 2020). Such ramps are often associated with the gradual approach toward reward or sensory feedback via stimuli that update about the prediction of impending reward (Kim et al., 2020; Mikhael et al., 2022) and, as in our hands, are more readily observed in OC compared with PC (Guru et al., 2020; Song and Lee, 2020; Hamid et al., 2021). However, in our experiment, the amount of sensory feedback (cue) and the distance of the animals to reward (food magazine) remain stable (from cue onset until cue offset) in both OC and PC animals. Thus, it is possible that multiple sequential cues (until reward delivery) induce a ramp in both OC and PC animals, but in the absence of such scenarios, only OC animals exhibit a ramp. Another inconsistency with previous reports is that we find remarkable stability of dopamine ramps across 14 d of behavioral training, whereas others have suggested and reported that ramps fade with extended training when task performance becomes asymptotic (Collins et al., 2016; Guru et al., 2020; Song and Lee, 2020), potentially linking credit back to the rewarded action to guide reward learning (Howe et al., 2013; Collins et al., 2016; Hamid et al., 2016). A potential function that would require such stable dopamine ramps is the encoding of reward expectation (Howe et al., 2013; Mohebi et al., 2019). However, in our paradigm, OC and PC rats had the same reward expectation, but only OC animals exhibited ramps. Together, this favors another explanation, the anticipation of performing a rewarded action; dopamine ramps could support the motivation to perform operant actions for distal rewards. Indeed, many previous findings tie the mesolimbic dopamine system to motivation (Berridge, 2007; Salamone et al., 2007; Nicola, 2010; Panigrahi et al., 2015; Hughes et al., 2020), and, moreover, some studies support the idea that dopamine ramps are implicated in motivation (Wassum et al., 2012; Howe et al., 2013; Collins et al., 2016; Hamid et al., 2016; Mohebi et al., 2019).

In summary, our findings suggest that sustained dopamine release during presentation of a reward-predicting cue can be driven by action anticipation by means of modulating a putative ramp-preceding dopamine RPE signal (which is a precondition to the dependent ramp). These findings shine light on how the mesolimbic dopamine system integrates reward- and action-related functions associated with the pursuit of rewarding outcomes in a temporally distinguishable manner and provide new insight into the nature and function of sustained or ramp-like dopamine release, which may embody an intermediate between learning and action, conceptually related to the motivation to generate a reward-achieving action.
